# Refractory fistula of bladder repaired with transurethral cystoscopic injection of *N*‐butyl‐2‐cyanoacrylate

**DOI:** 10.1002/iju5.12130

**Published:** 2019-11-07

**Authors:** Sakurako Mukai, Shunsuke Shinmei, Masayuki Muto, Tomoya Hatayama, Hiroyuki Shikuma, Shunsuke Miyamoto, Shinsuke Fujii, Kousuke Sadahide, Yohei Sekino, Keisuke Hieda, Shogo Inoue, Tetsutaro Hayashi, Jun Teishima, Akio Matsubara

**Affiliations:** ^1^ Department of Urology Hiroshima University Graduate School of Biomedical Sciences Hiroshima Japan

**Keywords:** bladder fistula, cystoscopic injection, *N*‐butyl‐2‐cyanoacrylate

## Abstract

**Introduction:**

Refractory fistulas of the bladder are not rare, but they can rarely be closed naturally. Bladder fistulas can be treated in various ways. We report the case of an old woman who had a refractory fistula of the bladder that was able to be repaired with transurethral cystoscopic injection of *N*‐butyl‐2‐cyanoacrylate.

**Case presentation:**

For decades after being treated for cervical cancer in 1970s, the woman frequently suffered from fevers. A computed tomography scan showed pelvic abscess at the left side of her bladder, and cystography showed urine leakage at the wall. Thus, we diagnosed her with a pelvic abscess due to a bladder fistula after radiation. Then, we treated her with drainage, antibiotic agents, and *N*‐butyl‐2‐cyanoacrylate. After that, she no longer had fevers, and cystography showed no leakage of urine.

**Conclusion:**

This result indicates transurethral cystoscopic injection of *N*‐butyl‐2‐cyanoacrylate may treat bladder fistulas safely, minimally invasively, and quickly.

**Abbreviations & Acronyms**
CT: computed tomography
NBCA: *N*‐butyl‐2‐cyanoacrylate

**Keynote message**

We report the case of an old woman who had refractory fistula of the bladder that was able to be repaired with transurethral cystoscopic injection of NBCA. Transurethral cystoscopic injection of NBCA may treat bladder fistulas safely and minimally invasively.

## Introduction

Refractory fistulas of bladder are not rare, but they can rarely be closed naturally. There are various ways to treat bladder fistulas, but few are safe, minimally invasive, and quick. We report the case of an old woman who had a refractory fistula of the bladder that was able to be repaired with transurethral cystoscopic injection of NBCA (Histoacryl^®^; B/Braun, Tuttlingen, Germany). NBCA has only a slight complication and is a quick way to treat bladder fistulas. Therefore, we report a case of transurethral cystoscopic injection of NBCA.

## Case presentation

A 71‐year‐old woman repeatedly suffered from fevers. She had previously undergone surgery and radiation for cervical cancer in 1970s, which left her with ureteral stenosis due to radiation and pelvic abscess because of a bladder fistula after radiation, so we implanted nephrostomy tubes to both her kidneys. High fevers were the only after‐effect that generally appeared. Abnormal laboratory test values included white blood cell 9.79 × 10^3^/μL, C‐reactive protein 23.8 mg/dL, urea nitrogen 13.9 mg/dL, and creatinine 0.95 mg/dL. Both nephrostomy tubes’ urine volumes showed no problem. Moreover, both nephrostomy tubes’ urine cultivations were identified as *Pseudomonas aeruginosa* and *Enterococcus faecalis*, but the blood's cultivation was identified as negative.

**Figure 1:**
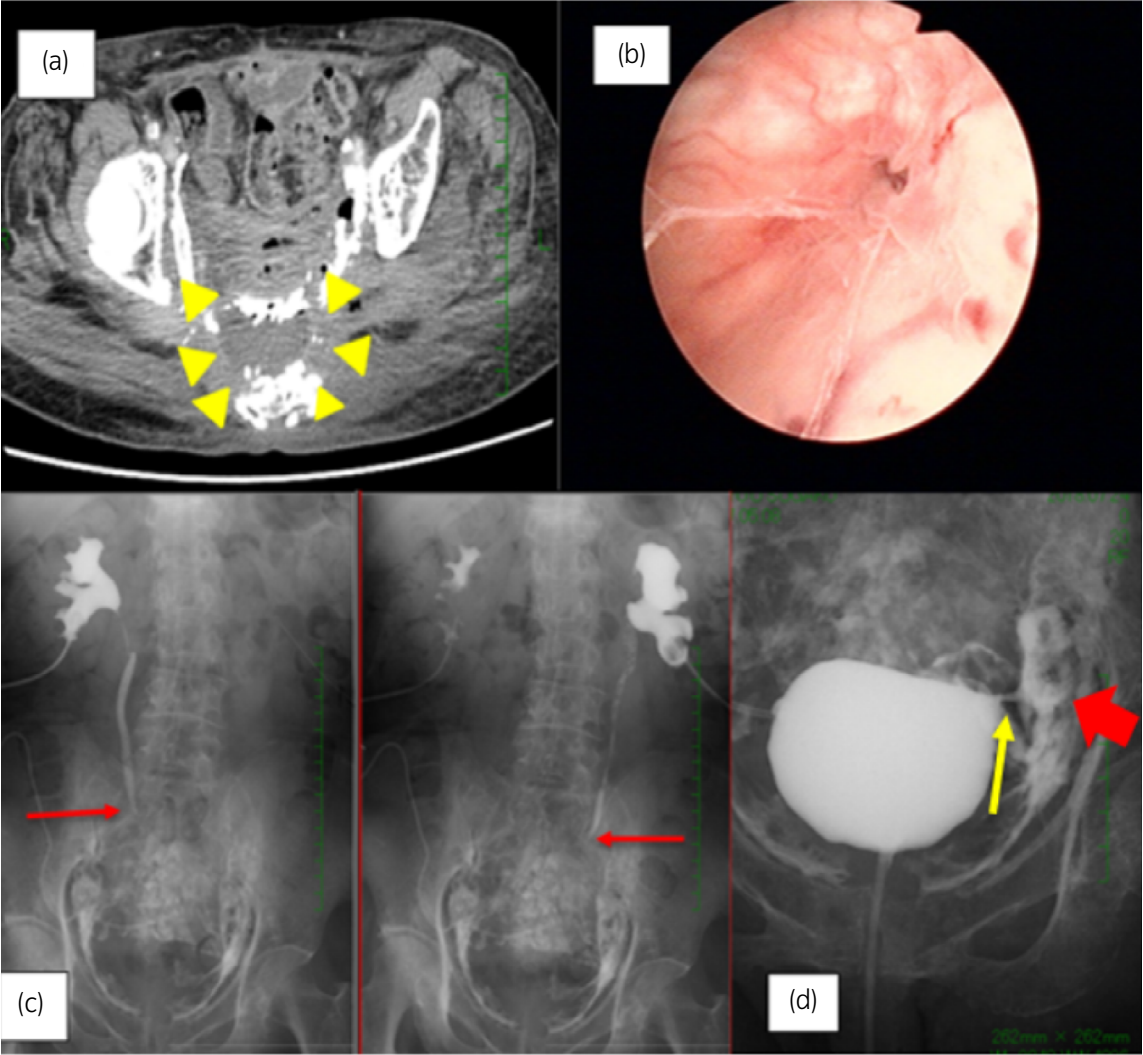
(a) Contrast‐enhanced CT scan identifying liquid in the ventral of the patient's sacrum bone (yellow arrowheads) and calcification around the liquid. This suggests calcification is due to radiation. (b) Cystoscope image indicating a fistula of about 2 mm at the left cyst wall. (c) Antegrade pyelography showing we cannot confirm the contrast under both sides of the mid‐ ureters and in her bladder (red arrow). (d) Cystography showing urine leakage from her left cyst wall (yellow arrow) to the abscess cavity (red arrow).

A contrast‐enhanced CT scan showed liquid in the ventral of her sacrum bone and calcification around the liquid. This suggested calcification was due to radiation (Fig. 1a). A cystoscope indicated a fistula of about 2 mm at the left bladder wall (Fig. 1b). Antegrade pyelography showed we could not confirm the contrast under both sides of her mid‐ureters and in her bladder (Fig. 1c). Cystography showed urine leakage from her left bladder wall to the abscess cavity (Fig. 1d). Moreover, gastrointestinal and gynecological examinations were performed, which confirmed that the fistula was in the bladder only. These findings led to a diagnosis of a pelvic abscess caused by a bladder fistula after radiation.

**Figure 2:**
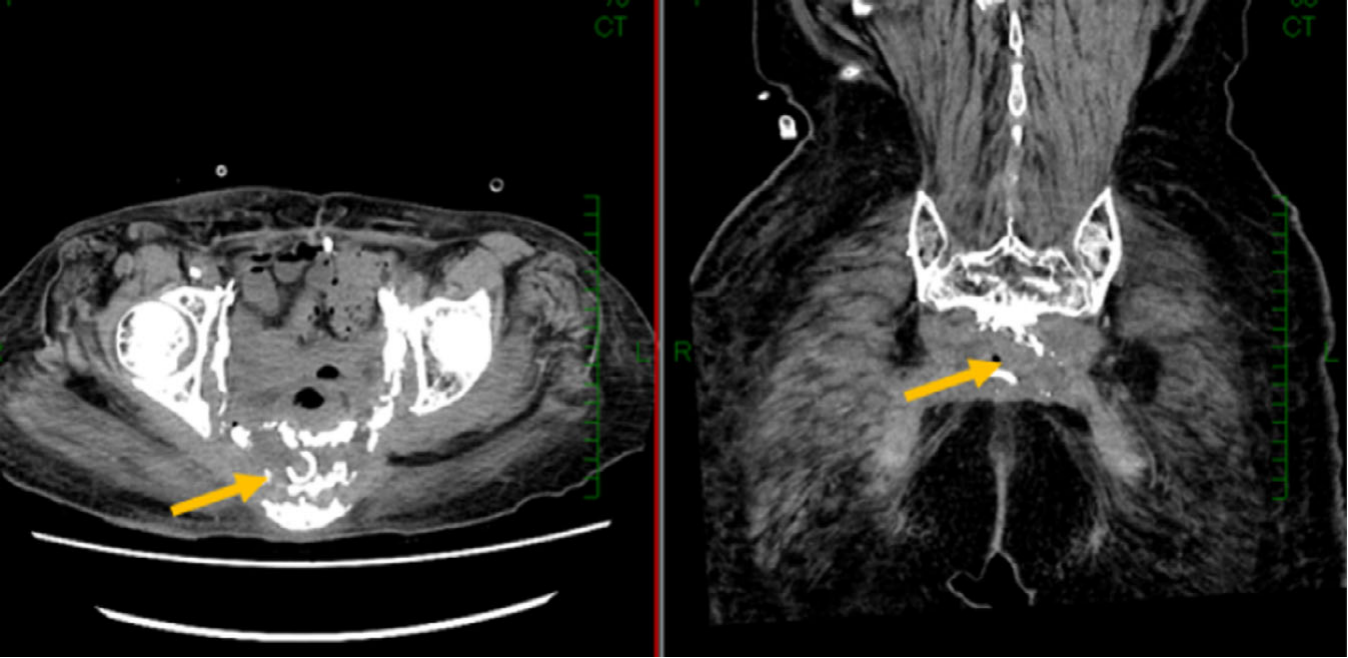
CT scans showing we performed CT‐scan guided drainage at the site of the retroperitoneal abscess identified in Figure 1a (yellow arrowheads).

First, we started antibiotic medication and drained the pelvic abscess under the CT‐scan guide (Fig. 2). We used only meropenem, because she had a lot of resistant bacteria. Then, the pelvic abscess drainage was identified as *Fusobacterium necrophorum*, and *Bacteroides thetaiotaomicron*.

**Figure 3:**
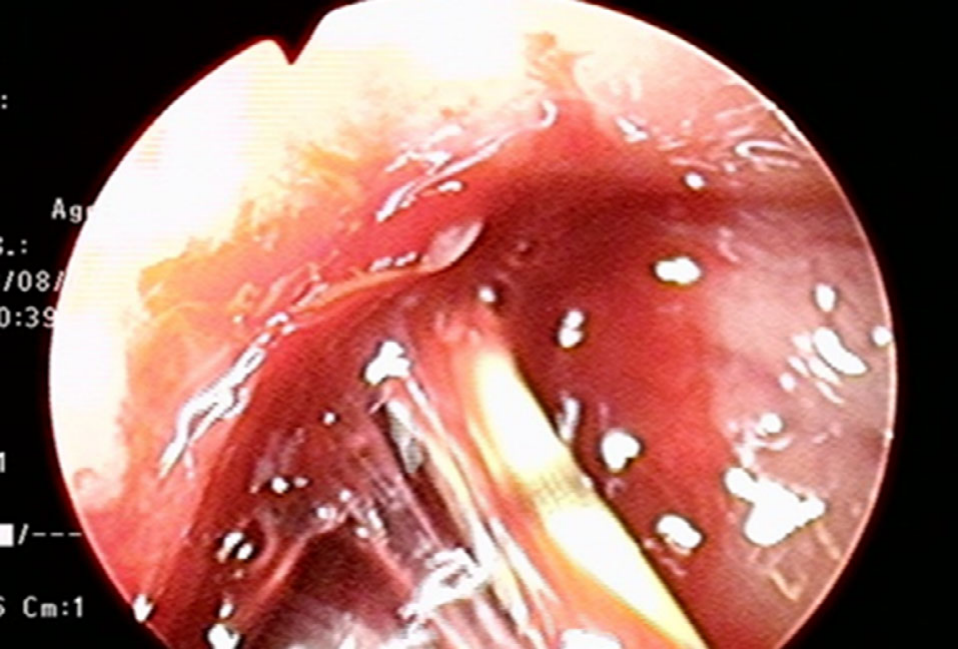
An open‐ended 5‐Fr ureteral catheter was advanced over a hydrophilic guide wire in the patient's left cyst wall. NBCA of 1 mL was mixed with iodized oil of 1 mL. After that, we injected the NBCA into the fistula and rapidly withdrew the ureteral catheter.

**Figure 4:**
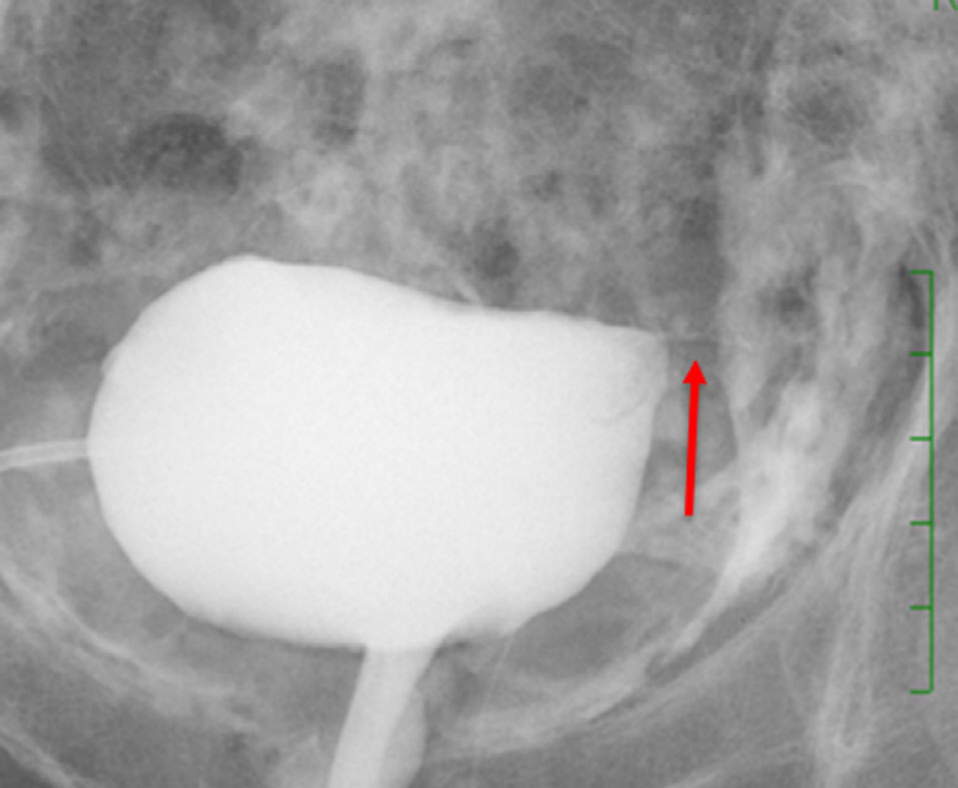
This cystography shows no leakage after injection of NBCA (red arrow). This figure is more difficult to understand than the actual image. Since NBCA was used as a mixture of contrast agents, it looks as though the occlusion site is angiographically visible and a fistula remains.

Second, we observed that her fevers were reduced after 10 days of medical treatment and her inflammatory reaction was reduced after 20 days, so we closed her bladder fistula with transurethral cystoscopic injection of NBCA. This procedure was in accordance with the Evaluating Committee for New Unapproved Drugs of Hiroshima University (approval number: 30). Specifically, a 5‐Fr ureteral catherter that we filled with saline solution was advanced over a hydrophilic guide wire in the left bladder wall. NBCA of 1 mL was mixed with iodized oil (Lipiodol^®^; Guerbet, Paris, France) of 1 mL. After that, we injected NBCA into the fistula and rapidly withdrew the ureteral catheter (Fig. 3). After a few minutes, we performed cystography, which showed no leakage (Fig. 4). We closed her bladder fistula with transurethral cystoscopic injection of NBCA while the drain was in the abscess. This was because, after the closure, the drain was left in place in order not to make the abscess cavity a closed space.

Finally, the drain was withdrawn after confirming that the abscess cavity had shrunk after 30 days of medical treatment. The patient had no fevers after the drain was withdrawn.

## Discussion

We sometimes have to conduct emergency surgery when a patient has serious symptoms or an intraperitoneal fistula. However, this fistula is difficult to close because its borders are fibrotic and poorly vascularized, and any attempt to close it in a usual way is doomed to failure.^1^ For example, some reports demonstrate vesicovaginal fistula repair with an omental wrap.^1^ However, although the omentum can certainly close the fistula, the operation takes a long time and is highly invasive.^2^ On the other hand, if a patient does not need emergency surgery, we can use transurethral cystoscopic injection of NBCA. NBCA is often used as an instant glue and a tissue‐adhesive material that has been used to close small surgical wounds.^3^

The best environment in which to use NBCA has not been clarified. However, because NBCA polymerizes when it comes into contact with moisture, it can also be used in water.^4^ In the case presented here, we performed a transurethral injection of NBCA without urine in the bladder. The reason for this is to prevent urine from displacing the occlusion position, but occlusion is possible even in the presence of urine. In addition, the suitable adaptation size of NBCA has not been clarified. In a previous paper, fistulas smaller than 2 mm were more easily repaired than fistulas bigger than 2 mm, and the closing success rate was 71% for fistulas smaller than 2 mm fistulas but 33% for fistulas bigger than 2 mm.^5^ Injections almost always fail to close fistulas that have diameters wider than 1 cm.^6^

From these results, we think the fistula connecting the retroperitoneal space and about 2 mm in size seems to be the best candidate for transurethral injection of NBCA. Even if the fistula does not meet these conditions like in the case mentioned above, since transurethral injection of NBCA is less invasive, it may be able to be tried once unless the case is urgent.

NBCA cannot contrast by itself, so we have to mix it with iodized oil when we use NBCA under an X‐ray. We often use NBCA mixed with iodized oil at a 1:1~2 ratio. We had difficulty injecting a part of peristalsis perfectly.

In our experience, we need a lot of skill when we use NBCA.^3^ On the other hand, transurethral cystoscopic injection of NBCA to seal a bladder fistula represents a better alternative than open surgical repair because, the side‐effect is only the escape of the polymer. In other words, the complication is only reopening of the fistula, but a bladder fistula can be sealed semi‐permanently in many cases.

## Conclusion

This results indicate that transurethral cystoscopic injection of NBCA may treat bladder fistulas safely, minimally invasively, and quickly.

## Conflict of interest

The authors declare no conflict of interest.
